# Neurotensin Is a Lipid-Induced Gastrointestinal Peptide Associated with Visceral Adipose Tissue Inflammation in Obesity

**DOI:** 10.3390/nu10040526

**Published:** 2018-04-23

**Authors:** Ilaria Barchetta, Flavia Agata Cimini, Danila Capoccia, Laura Bertoccini, Valentina Ceccarelli, Caterina Chiappetta, Frida Leonetti, Claudio Di Cristofano, Gianfranco Silecchia, Marju Orho-Melander, Olle Melander, Maria Gisella Cavallo

**Affiliations:** 1 Department of Experimental Medicine, Sapienza University of Rome, 00161 Rome, Italy; ilaria.barchetta@uniroma1.it (I.B.); flaviaagata.cimini@uniroma1.it (F.A.C.); danila.capoccia@uniroma1.it (D.C.); laura.bertoccini@hotmail.com (L.B.); valentina.ceccarelli1@gmail.com (V.C.); frida.leonetti@uniroma1.it (F.L.); 2 Department of Medical-Surgical Sciences and Bio-Technologies, Sapienza University of Rome, 00161 Rome, Italy; caterina.chiappetta@uniroma1.it (C.C.); claudio.dicristofano@uniroma1.it (C.D.C.); gianfranco.silecchia@uniroma1.it (G.S.); 3 Department of Clinical Sciences, Lund University, 20502 Malmoe, Sweden; marju.orho-melander@med.lu.se (M.O.-M.); olle.melander@med.lu.se (O.M.)

**Keywords:** proneurotensin, adipose tissue inflammation, obesity, lipids

## Abstract

Neurotensin (NT) is a 13-amino acid peptide localized in the neuroendocrine cells of the small intestine, which promotes fat absorption and fatty acids translocation in response to lipid ingestion. NT-knock-out mice fed with a high-fat diet are protected from obesity, fatty liver, and the development of insulin-resistance. In humans, higher plasma levels of pro-NT, which is the stable circulating precursor of NT, predict obesity, type 2 diabetes (T2D), and cardiovascular disease. In obesity, the presence of visceral adipose tissue (VAT) inflammation leads to unfavorable metabolic outcomes and is associated with the development of T2D and non-alcoholic fatty liver disease (NAFLD). In this study, we investigated the relationship between plasma pro-NT levels and the presence of VAT inflammation in biopsies from 40 morbidly obese subjects undergoing bariatric surgery. We demonstrated that higher proNT levels are significantly associated with greater macrophages infiltration, HIF-1α, WISP-1, and UNC5B expression in VAT (all *p* < 0.01) due to the diagnosis of T2D and NAFLD. The overall results show that, in obesity, pro-NT is a biomarker of VAT inflammation and insulin-resistance. Additionally, NT may be involved in the development of dysmetabolic conditions likely mediated by increased gut fat absorption and the presence of a proinflammatory milieu in the adipose tissue.

## 1. Introduction

Neurotensin (NT) is a 13-amino acid peptide secreted by the neuroendocrine cells of the small intestine in response to fat ingestion, which facilitates fatty acid absorption through the gut in relation to food lipid content [1]. Moreover, NT acts as a neurotransmitter in the central nervous system by regulating ghrelin and leptin-associated pathways that mediate satiety and food ingestion in the lateral hypothalamic area [2,3,4]. Very recently, we demonstrated the existence of a positive association between NT and leptin levels in the bloodstream, which makes the hypothesis that NT modulates leptin concentration in periphery through mechanisms involving gut fat absorption more plausible [5]. Several experimental models show that NT mediates the development of fatty liver disease and obesity [6,7,8] while, in humans, large epidemiological studies demonstrate that higher circulating levels of pro-neurotensin (pro-NT), which is the stable NT precursor released in equimolar amount with NT, predict obesity, type 2 diabetes (T2D), and cardiovascular disease [9,10]. Obesity-associated diseases such as insulin-resistance, T2D, and metabolic syndrome (MS) have been associated with adipose tissue (AT) dysfunction occurring in conditions of chronic excessive caloric intake [11]. Therefore, the nutrients overload impairs the physiological plasticity of adipocytes, which results in fatty acid spill over, insufficient neovascularization, necrosis, and recruitment/activation of several immune cells such as macrophages, dendritic cells, and lymphocytes. Moreover, in obesity, a significant downregulation of the VAT expression of genes involved in lipid metabolism has been shown. This likely describes a condition of plateau in the storage capacity of adipocytes, which is not capable to further synthetize fatty acids [12]. The overall result is chronic AT inflammation, which leads to systemic low-grade inflammation, increased circulating fatty acids concentration, and insulin-resistance and aberrant fat deposition in the liver [13,14,15]. NT and its receptor NTR1 have been recently shown to be over-expressed in colitis-associated visceral AT (VAT) inflammation [16,17]. NT also stimulates the preadipocyte-dependent macrophage migration in vitro through a mechanism involving the IL-6 release [17]. The overall data, therefore, points towards a direct role of NT in regulating macrophage function, which induces inflammatory pathways leading to VAT dysfunction. However, whether NT is associated with VAT inflammation in obesity and metabolic disease has not yet been investigated. Therefore, the aim of this study was to investigate the relationship between systemic NT and VAT inflammation in morbidly obese individuals with and without T2D.

## 2. Materials and Methods

### 2.1. Population

For this study, we recruited 40 obese individuals to bariatric surgery referring to the Diabetes and Endocrinology outpatient clinics, Sapienza University of Rome, Italy, for pre-operative metabolic evaluations. Study participants were both males and females aged between 25 and 65 years, had clinical indication to sleeve gastrectomy, and met the following inclusion/exclusion: no history of current or past excessive alcohol drinking (daily consumption of alcohol >30 g/day in men and >20 g/day in women), negative tests for hepatitis B and C, absence of history and findings consistent with cirrhosis and other causes of liver diseases (autoimmune hepatitis, hemochromatosis, Wilson’s disease), and no treatment with drugs potentially inducing liver steatosis (e.g., corticosteroids, oestrogens, methotrexate, tetracycline, calcium channel blockers, and amiodarone). All the study participants signed a written informed consent before undergoing all the study procedures. The study protocol has been reviewed and approved by the local Ethics Committee and conducted in conformance with the Helsinki Declaration.

### 2.2. Clinical and Laboratory Assessments

Study population underwent complete clinical workup including height, weight, and waist circumference measurement and body mass index calculation (BMI; weight (kg) × height (m^2^)), systolic (SBP) and diastolic (DBP) blood pressure were assessed after 5 min of resting, three measurements were taken, and the average of the second and third measurements was recorded and used for the analyses. Overnight fasting blood sampling was performed in all the study participants for assessing blood glucose (FBG, mg/dL), glycosylated hemoglobin (HbA1c, %—mmol/mol), total cholesterol (mg/dL), high-density lipoprotein cholesterol (HDL, mg/dL), triglycerides (mg/dL), aspartate aminotransferase (AST, IU/L), alanine aminotransferase (ALT, IU/L), and creatinine (mg/dL) by centralized standard methods. Fasting blood insulin (FBI, l U/mL) was measured by radioimmunoassay (ADVIA Insulin Ready Pack 100; Bayer Diagnostics, Milan, Italy) with intra-assay and inter-assay coefficients of variation <5%. The low-density lipoprotein (LDL) cholesterol value was calculated by the Friedwald formula. Insulin resistance and secretion were estimated by calculating the HOMA-IR and HOMA-β secretion indexes, respectively. Diabetes mellitus was defined by the ADA criteria [18] and the metabolic syndrome was diagnosed based on the modified NCEP-ATPIII criteria [19]. Circulating concentration of pro-NT, the stable NT precursor fragment released in equimolar amounts relative to NT, was measured on plasma frozen immediately after separation and stored at −80 °C. Pro-NT was measured using a chemiluminometric sandwich immunoassay to detect pro-NT amino acids 1–117, which was described previously [20]. The analytical assay sensitivity was 4.8 pmol proNT/L. The inter assay (10 assay runs) coefficient of variability was 6.2% at 48 pmol proNT/L and 4.1% at 191 pmol/L. 

### 2.3. Histological and Immunohistochemical Analysis

Histological examinations were performed on omental biopsies (~1 cm^3^) obtained from patients undergoing laparoscopic sleeve gastrectomy for clinical purposes. VAT biopsies were fixed with 10% buffered formalin for 24 h and then paraffin-embedded (FFPE). Hematoxylin and eosin (H&E) stained sections were performed for histological evaluation and, for all the samples, a consecutive section (3 µm) was stained with Masson’s trichrome and used for analysis of fibrosis. Two pathologists, blinded to the experimental protocol, performed the analyses. To evaluate the infiltration of macrophages, we performed immuno-histochemical analysis using CD68 monoclonal antibody (clone M0876, 1:100; Dako, Carpinteria, CA, USA) and evaluated changes in micro-vessel density by CD34 immunostaining (clone QBEnd/10; Leyca Biosystem, Newcastle, UK). From paraffin tissue blocks, AT consecutive tissue sections (2 µm) were cut and the sections were deparaffinized and rehydrated. Endogenous peroxidase activity was blocked with 3% hydrogen peroxide and antigen retrieval was made by boiling them in citrate buffer (0.01 mol/L, pH 6) with microwaves (750 W). The sections were incubated for 1 h at room temperature (RT) with primary antibodies and universal LSAB2 System-HRP (Dako, Carpinteria, CA, USA) was used to label the primary antibodies. The samples were then washed with TBS buffer and incubated with freshly prepared DAB + substrate–chromogen buffer at room temperature. After gently rinsing with ddH_2_O, the slides were counterstained with hematoxylin and mounted with permanent mounting media. Both positive and negative internal and external controls were used in each experiment. Results were expressed through a semi-quantitative scale by evaluating the percentage of CD68 positive cells in five random fields (40×) of each sample and then the mean value was used to define the percentage of positive cells per sample. The stained sections were first screened at low power (×10) to determine the areas of most intense staining for CD34. Then the blood vessel counting was performed under ×40 magnification in five random fields. 

### 2.4. Gene Expression Analysis—RealTime PCR

Total RNA from FFPE samples was extracted using RecoverAll™ Total Nucleic Acid Isolation Kit for FFPE (ThermoFisher Scientific, Waltham, MA, USA), according to the manufacturer’s instructions. Purity and quantity of RNA were confirmed by NanoDrop ND-1000 Spectrophotometer (ThermoFisher Scientific, Waltham, MA, USA). RNA was reverse transcribed into cDNA with High-Capacity cDNA Reverse Transcription Kit (Thermo Fisher Scientific, Waltham, MA, USA). PCR products of human *NTN1*, *UNC5B*, *CAV1*, *IL8*, *MIP1A*, *MIP2*, *TIMP1*, *GZMB*, *CASP3*, *CASP7*, *PARP1*, *HIF1A*, and *WISP1* were detected by using gene-specific primers and probes labeled with reporter dye FAM. *GAPDH* was used as an internal standard, which yielded a predicted amplicon of 171 bp. TaqMan real-time quantitative PCR was performed on an ABI PRISM 7500 Fast Real-Time PCR System (Applied Biosystem, Foster City, CA, USA). PCR reactions were carried out in triplicate on 96-well plates with 10 L per well using 1× TaqMan Master Mix. After 2-min incubation at 50 °C and 10 min at 95 °C, the reactions continued for 40 cycles at 95 °C for 15 s and 60 °C for one minute. At the end of the reaction, the results were evaluated using the ABI PRISM 7500 software (Applied Biosystem, Foster City, CA, USA). The cycle threshold (*C*t) values for each set of 3 reactions were averaged for all subsequent calculations. The 2^−Δ*C*t^ method was used to calculate relative changes in gene expression.

## 3. Results

In the whole study population, mean ± SD plasma pro-NT concentration was 178.6 ± 85 pmol/L [median (25–75°): 159.4 (118–216.9) pmol/L] (Table 1) and positively correlated with greater age (r = 0.43, *p* = 0.004), (triglycerides r = 0.39, *p* = 0.012), (HbA1c r = 0.40, *p* = 0.012), (FBI r = 0.38, *p* = 0.02), with the diagnosis of T2D (r = 0.33, *p* = 0.039), and the presence and severity of NAFLD evaluated by the NAS score (r = 0.41, *p* = 0.01, r = 0.36, *p* = 0.023; respectively). In contrast, no association was found between pro-NT, systemic blood pressure, total HDL cholesterol, LDL cholesterol, transaminases, waist circumference, and BMI. No significant difference was observed between circulating proNT levels in male and female participants (181.6 ± 87.9 vs. 182.5 ± 90.6 pmol/L, respectively, *p* = 0.97). All the bivariate analyses are shown in Table 2. 

When considering glucose tolerance, patients with T2D showed significantly higher plasma proNT levels than non-diabetic individuals (160.9 ± 87.9 vs. 280.6 ± 133.7 pmol/L, *p* = 0.02). Moreover, the study population was further stratified according to the glycemic state (normo-glycaemia, impaired fasting glucose (IFG)/impaired glucose tolerance (IGT), and T2D) and we observed that plasma pro-NT levels significantly increased throughout classes of impaired glucose metabolism (normo-glycaemic individuals: 155 ± 53.2 pmol/L vs. IFG/IGT: 176.6 ± 90.8 pmol/L vs. T2D: 280.6 ± 133.7 pmol/L; *p* < 0.01, ANOVA test applied). Finally, the multivariate linear regression analysis showed that greater age and triglycerides concentration represented the main predictors of increased circulating proNT levels after adjusting for BMI, gender, and all the parameters significantly associated with the bivariate correlation analyses (R = 0.55, R^2^ = 0.30; *p* = 0.014). 

Higher pro-NT levels were significantly associated with features of VAT inflammation explored by both immunohistochemistry and VAT gene expression analyses. 

**Figure 1:**
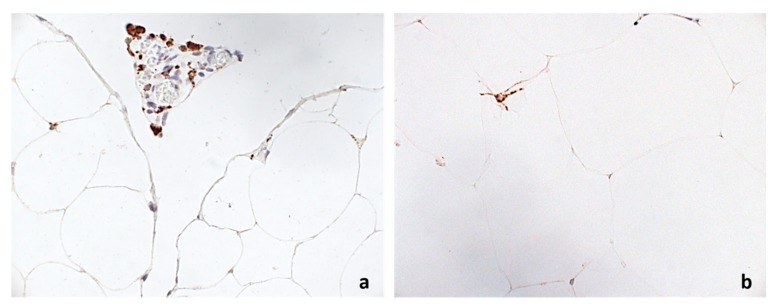
Immunohistochemical expression of CD68 in: VAT of a patient with high proNT ((**a**), 400×), and VAT of a patient with low proNT ((**b**), 400×).

In our study population, median (range) CD68+ expression was 2 (0–10)% and median (range) CD34+ expression was 2 (1–7)%. Greater circulating pro-NT concentration, as expressed by pro-NT above the median value, was associated with significantly higher CD68+ (CD68+ > median expression, OR 4.2 (95% C.I.: 2.4–7.2), *p* < 0.001; Figure 1) and lower CD34+ expression (CD34+ < median expression; OR 0.2 (95% C.I.: 0.05–0.87), *p* = 0.03). 

Higher pro-NT levels also correlated with increased HIF-1α (r = 0.41, *p* = 0.011), WISP-1 (r = 0.37, *p* = 0.022), and UNC5B (r = 0.42, *p* = 0.009) expression in VAT (see Table 3). 

## 4. Discussion

The main finding of this study is the relationship between higher pro-NT levels and histological evidence of VAT inflammation in morbidly obese patients. Furthermore, in our study population, circulating pro-NT also correlated with the presence of T2D and NASH, which represents a possible biomarker of metabolic impairment in obesity. The existence of an independent association between higher proNT levels and the presence and severity of NAFLD/NASH has been very recently demonstrated by our group in a population of obese and non-obese individuals with and without T2D [21]. 

In the present study, pro-NT levels positively correlate with triglycerides concentration, which aligns with evidence from experimental models. NT administration was shown to directly modulate lipid metabolism in rats [22].

VAT inflammation and the subsequent metabolic dysfunction are implicated in the development of obesity-related complications such as systemic low-grade inflammation, insulin-resistance, and T2D. In particular, VAT dysfunction has been recognized as determining ectopic fat distribution and NAFLD in humans due to the aberrant FFAs and adipokine release from an unhealthy adipose cell tissue under a stressful chronic caloric overload. However, whether VAT dysfunction is a requirement across the lifespan of an obese individual has not been yet elucidated and neither genetic or environmental factors potentially determining VAT dysfunction in obesity have been identified so far. Additionally, the overall data on an integrated action of NT in regulating food intake and energy balance may provide a pathophysiological explanation to the excess of cardiovascular risk associated with higher pro-NT levels reported in several epidemiology studies [9,10]. Furthermore, NT exerts impact other relevant factors controlling body weight such as leptin and ghrelin [4]. In addition, several metabolic mediators might be potentially involved in NT regulation [23].

Therefore, increased pro-NT may significantly promote lipids absorption by inducing both directly and indirectly, which includes favoring body weight gain, fat accumulation, and dysmetabolism. 

Despite the cross-sectional design of this study, it does not allow to establish the causal relationship of our findings. It is possible to hypothesize that NT is involved in the development of dysmetabolic conditions likely through increased gut fat absorption and the induction of a pro-inflammatory milieu in the adipose tissue. Moreover, since increased plasma pro-NT levels are associated with both VAT inflammation and NAFLD [21], pro-NT measurement may represent a potential tool for a more accurate risk stratification in obese patients as well as a novel success criterion for metabolic and bariatric surgery [24].

Longitudinal studies are warranted in order to explore the potential role of pro-NT as a determinant of VAT inflammation and related-metabolic diseases in obesity.

## Figures and Tables

**Table 1:** Clinical and biochemical characteristics of the study population.

Parameter	Mean ± Standard Deviation Rate (%)	Median (25°–75° Percentile)
Age (years)	43.7 ± 9.6	42 (38–49)
Gender (M%)	24%	-
BMI (kg/m^2^)	43.6 ± 5.6	42.2 (40–47.4)
Waist circumference (cm)	128 ± 11.4	126 (126–135.5)
SBP (mmHg)	128.3 ± 16.1	130 (120–136)
DBP (mmHg)	84.8 ± 18.2	80 (80–85)
Total Cholesterol (mg/dL)	196 ± 32.4	194.5 (176.2–207)
HDL (mg/dL)	48.2 ± 10.4	48 (39–55)
LDL (mg/dL)	120.3 ± 29.7	120 (120–137)
Triglycerides (mg/dL)	129.5 ± 44.9	127 (127–160)
FBG (mg/dL)	104 ± 22.4	95 (90–120)
HbA1c (%—mmol/mol)	5.9 ± 1	5.3 (5.1–5.8)
FBI (µU/L)	16.8 ± 15.1	11.5 (9.7–16.6)
HOMA-IR	4.5 ± 4.7	2.8 (2.2–4)
HOMA-β%	164 ± 120	133.5 (67.8–212)
Pro-NT (pmol/L)	178.6 ± 85	159.4 (118–216.9)
T2D (%)	15%	-
MS (%)	80%	-
NAFLD (%)	50%	-
NASH (%)	25%	-
Therapy with antidiabetic agents	15%	-
Therapy with statins	92%	-
Therapy with anti-hypertensive medications	73%	-

**Table 2:** Pro-NT-Bivariate correlation analyses (Pearson’s coefficient, * Spearman’s coefficient, pro-NT is considered a continuous variable).

Parameter	Correlation Coefficient	*p*-Value
Age	0.43	0.004
Gender (M/F)	0.02 *	0.89
BMI	0.31	−0.16
Waist circumference	−0.16	0.31
FBG	0.07	0.67
FBI	0.38	0.02
HbA1c	0.40	0.012
Total Cholesterol	0.02	0.89
HDL	−0.04	0.78
LDL	0.02	0.88
Triglycerides	0.39	0.012
AST	−0.08	0.61
ALT	0.04	0.80
T2D yes/no	0.33 *	0.039
NAFLD yes/no	0.41 *	0.01
NAS score	0.36 *	0.023

**Table 3:** Bivariate correlation analyses between circulating pro-NT levels and VAT gene expression (Spearman’s coefficient).

Gene	Correlation Coefficient	*p*-Value
NTN1	−0.11	0.50
UNC5B	0.42	0.009
CAV1	0.11	0.50
IL8	−0.08	0.61
MIP1A	0.08	0.61
MIP2	0.11	0.50
TIMP1	0.15	0.38
GZMB	−0.13	0.43
CASP3	−0.02	0.9
CASP7	0.09	0.58
PARP1	0.16	0.43
HIF-1α	0.41	0.011
WISP1	0.37	0.022
